# Clinical Lectures on Diseases of the Eye

**Published:** 1865-10

**Authors:** E. L. Holmes

**Affiliations:** Chicago, Lecturer on Diseases of the Eye and Ear in Rush Medical College, and Surgeon of the Chicago Charitable Eye and Ear Infirmary


### CLINICAL LECTURES ON DISEASES OF THE EYE.

By E. L. HOLMES, M. D., of Chicago,

*Lecturer on Diseases of the Eye and Ear in Rush Medical College, and Surgeon of the Chicago Charitable Eye and Ear Infirmary.*

### THE CRYSTALLINE LENS.

GENTLEMEN :

The crystalline lens is double convex in form, more convex posteriorly than anteriorly, of perfect transparency and great elasticity. It is about four lines in diameter, and varies in different individuals from one and a half to two and a quarter lines in thickness at its antero-posteri or axis.

The lens is situated behind the iris, its axis nearly coinciding with a line drawn through the centre of the cornea and of the globe itself. The pupillary edge of the iris rests in contact with its anterior convex surface ; but the periphery of the iris is slightly separated from the lens. The space thus formed is termed by some authors the posterior chamber Other writers apply the term posterior chamber to the portion of the eye, posterior to the lens ; the anterior chamber being the space occupied by the aqueous humor.

The posterior surface of the lens is embeded in a cup-shaped depression in the vitreous humor, and adheres slightly to the delicate hyaloid membrane, which surrounds this humor and which is firmly united with the Zonula of Zinn near the anterior border of the retina. As different authorities vary somewhat in their description of the zonula, and as the description in my last lecture may appear somewhat obscure, I will recall your attention to the subject.

At the ora serrata the retina loses its nervous structure and passes forward to the ciliary processes as a delicate almost structureless membrane. The hyaloid is so intimately united with this membrane, that it is difficult with the highest power of the microscope to trace each layer separately.

This membrane subdivides into two layers,—the true zonula passing on to the anterior part of the capsule of the lens—and the posterior layer continuing forward as the hyaloid, to be attached to the posterior part of the capsule of the lens. The triangular space between these two lamina and the periphory of the lens is called the canal of Petit.

The lens, although held quite firmly in position by these attachments, may suffer considerable displacement forwards without injury. With certain incisėd or punctured wounds of the cornea, the aqueous humor may be discharged, and the iris and lens pressed forward in contact with the cornea. As the interior chamber becomes refilled they often recede to their normal position uninjured.

The relative position of the cornea, iris and lens is worthy of especial consideration. You will recollect that the cornea is about six lines in diameter, the iris somewhat more, its periphery extending slightly beyond that of the cornea, and that the diameter of the lens is about four lines. It will thus appear evident to you how an instrument, a cataract knife for instance, can be introduced into the eye a little posterior to the union of the cornea and sclerotic in such a way as to open the anterior chamber at its periphery, evacuate the aqueous humor and divide the ciliary processes, without injury to the lens. This is done in Hancock's operation to which I have already called your attention.

The lens is composed of two distinct portions, the capsule and its enclosed lenticular substance. The capsule is a perfectly transparent membrane, almost without a trace of cell structure. The anterior half is quite firm, offering a perceptible resistance when pressed by the point of an instrument. The posterior half is less firm. The lenticular portion is composed of a hard kernel surrounded by a soft cortical substance. The general structure of the lens may be best studied by first boiling it a few minutes in water. It will then be found that it is quite complex in structure, consisting of a large number of concentric layers which are composed of minute fibres. These fibres are six-sided, and arranged as represented in the plates before you. The arrangement of these layers and fibres is the same in the soft cortical substance and in the hard central kernel. The lens of fish and the lower animals after boiling may be studied with advantage, when one from the human eye is not at hand.

No nerves or vessels have been discovered in any portion of the lens. Nutrition is maintained by direct transfusion from the surrounding tissues, especially the iris and the choroid. Serious and persistent diseases of either of these organs are very liable to result in disturbed nutrition of the lens with more or less opacity. Molecular change apparently occurs with considerable rapidity, since slight punctures of the capsule may heal in a very short time, with scarcely a trace of opacity. Exudations from the iris in inflammation become very speedily united with the anterior capsule; the lens may also form adhesions with the cornea, in case of perforating ulcers of the latter, the aqueous humor being evacuated and the lens brought in contact with the latter. Slight wounds of the capsule may heal either with or without a visible cicatrix; penetrating injuries of the lens however are generally followed by opacity, softening and often with final absorption of the lenticular portions, the capsule being left as a delicate membrane floating behind the pupil. Concussions of the globe either from blows upon the eye or the head often produce opacity of the lens.

Attempts have been made to show that the lens after its destruction may be reproduced, but there are probably no observations sufficiently well authenticated to establish the fact.

In childhood the thickness of the lens at the axis is greater in proportion to the diameter than in advanced age. In childhood also the lens is much softer and elastic than subsequently. In infancy and old age the anterior convex surface is much nearer the cornea, making the anterior chamber much smaller than during other periods of life.

In advanced age the lens even when transparent acquires a yellowish cloudy appearance, which may be mistaken for cataract. Changes in the color of the choroid in the aged renders this cloudy appearance more perceptible. There is another peculiarity in the lens of many aged persons, which, like the rinkles in the face or the loss of pigment in the hair, can scarcely be considered as disease. An examination of a large number of aged individuals with the opthalmoscope will reveal in a certain proportion the presence of short dark lines arranged around the perpiphery of the lens. Since these lines are so often known to exist many years with scarcely any imperfection of vision, some pathologists have regarded them as one of the changes of old age and not necessarily as evidence of incipient cataract. This change in the appearance of the lens is analagous to the arcus senilis of the cornea. It in fact has been called the "arcus senilis of the lens."

The crystalline lens performs two important functions. With the cornea, aqueous and vitrious humors, it causes the rays of light from objects to converge and form an image within the eye.

It is also the instrument by means of which the eye is so readily adjusted to distinct vision at different distances.

